# Physiotherapy Protocol for Postoperative Intraosseous Rosai-Dorfman Disease of the Distal Femur: A Case Report

**DOI:** 10.7759/cureus.107224

**Published:** 2026-04-17

**Authors:** Sarthaki P Gaikwad, Nikhil V Bhoye, Fatima M Thoufeek, Hina B Jain, Rupali Shevalkar

**Affiliations:** 1 Department of Physiotherapy (Musculoskeletal Unit), K.J. Somaiya College of Physiotherapy, Mumbai, IND

**Keywords:** biopsy, bone neoplasms, histiocytosis, muscles, pain, physical therapy modalities

## Abstract

Rosai-Dorfman disease (RDD) is a rare, benign, non-neoplastic histiocytic proliferative disorder. Involvement of the distal femur with intra-articular extension is uncommon and can pose diagnostic challenges. Physiotherapy plays a critical role in postoperative recovery; however, structured rehabilitation guidelines for such cases remain limited in the literature. This case report describes a rare presentation of intraosseous RDD and highlights the clinical outcomes of a phased, impairment-based physiotherapy protocol following surgical excision and bone grafting.

The patient presented with gradually progressive left-sided knee pain that worsened over several months. Radiographic evaluation demonstrated a mixed lytic-sclerotic lesion in the distal femur with intra-articular extension. The patient underwent intra-articular curettage with allograft bone grafting; biopsy confirmed intraosseous RDD. She was referred for physiotherapy five weeks after immobilization. Postoperatively, she presented with restricted knee flexion range of motion (ROM) to 70°, extensor lag, fear of falling, swelling in the patellar region, a healed surgical scar, quadriceps and calf muscle atrophy, altered gait, pelvic drop during stance, reduced quadriceps, hamstring, and hip abductor strength (manual muscle testing (MMT) 3−), and impaired proprioception of the knee joint.

A structured, phase-wise physiotherapy program was initiated with the goals of protecting the graft, gradually restoring knee mobility, improving muscle strength, facilitating gait re-education, and enhancing proprioception. The patient demonstrated progressive improvement in knee range of motion, muscle strength, gait symmetry, and functional independence without complications.

This case underscores the importance of early, goal-oriented, phase-based physiotherapy following surgical management of intraosseous RDD of the distal femur. A structured rehabilitation protocol complemented surgical treatment, facilitating the recovery of mobility and function and helping to prevent long-term disability. This report contributes clinically relevant guidance to the limited literature on postoperative physiotherapy management in rare cases of RDD.

## Introduction

Rosai-Dorfman disease (RDD), also referred to as sinus histiocytosis with massive lymphadenopathy, is a rare, benign histiocytic proliferative disorder that was first described as a distinct clinical and pathological entity in 1969 [1,2]. The pathogenesis of RDD remains unclear, although infectious and immunological theories have been proposed.

The classic form of RDD is characterized by massive, painless, bilateral cervical lymphadenopathy and may be accompanied by constitutional symptoms such as fever and malaise [3]. Extranodal involvement is frequent, occurring in approximately 40% of patients, and may affect the skin, soft tissues, central nervous system, and upper respiratory tract. However, osseous involvement occurs in fewer than 10% of cases, and primary osseous RDD without associated lymphadenopathy is rare.

In cases of osseous involvement, lesions are usually osteolytic and may be accompanied by pain, swelling, or restricted joint movement, often simulating infection or malignancy [1]. Reported cases of osseous RDD often require surgical treatment, such as lesion excision and grafting, especially in cases involving painful or unstable bones. Postoperative results have shown pain relief and improvement in joint range of motion after proper surgical management [2].

Despite the growing number of case reports describing primary intraosseous RDD in long bones such as the tibia and ulna, there is a paucity of literature addressing structured postoperative rehabilitation or physiotherapy protocols in these patients. In particular, involvement of the distal femur as an isolated intraosseous lesion requiring postoperative functional rehabilitation remains scarcely documented in the literature [3]. Therefore, this case is unique in presenting a postoperative intraosseous RDD lesion at the distal end of the femur, with a structured physiotherapy protocol aimed at restoring knee function, strength, and mobility, an aspect that has not been adequately emphasized in previously published reports.

## Case presentation

Chief complaints

An 11-year-old female presented with complaints of difficulty bending the left knee post-surgery and fear of falling after a fall at home.

History of present illness

The patient was asymptomatic until December 2024, when she developed intermittent left posterior knee pain aggravated by prolonged walking. In April 2025, symptoms gradually progressed with difficulty walking and limited knee extension. Vitamin D evaluation showed a level of 6 ng/mL (normal range: 20-60 ng/mL), and supplementation provided temporary relief. In June 2025, the pain abruptly worsened with marked functional limitation and the development of a fixed flexion deformity, despite improved vitamin D levels (28 ng/mL). Orthopedic evaluation prompted radiographic and advanced imaging. In July 2025, the patient subsequently underwent intra-articular curettage with bone grafting, and biopsy findings were consistent with intraosseous RDD. Postoperatively, she was immobilized in a hip-to-knee Plaster of Paris slab for one month and remained non-weight-bearing. The cast was removed in a month, after which she was mobilized with a walker. Within 10 days, she progressed to independent ambulation with a mild residual limp. In August 2025, the patient experienced a slip while walking; however, no acute injury was identified. Subsequently, she developed a fear of falling and was referred for physiotherapy rehabilitation. The patient reported to the physiotherapy OPD five weeks postoperatively.

There was no relevant past medical or surgical history. Family and psychosocial histories were unremarkable and did not contribute to the present condition.

Clinical findings

Informed consent was obtained from the patient. On observation, swelling was present in the supra- and infrapatellar regions. A healed surgical scar was noted along the anterior midline of the patella. Muscle atrophy was evident in the quadriceps, vastus medialis oblique (VMO), and calf muscles.

Postural assessment revealed hip internal rotation, left genu valgum, internal tibial rotation, a pronated foot, and mild out-toeing. Gait analysis showed reduced weight-bearing on the left side with slight heel strike at initial contact, partial weight-bearing during loading response, and pelvic drop during terminal stance. Knee flexion was reduced during pre-swing and initial swing, with slight knee flexion in mid-swing and an inability to achieve full knee extension during terminal swing.

Motor assessment using a goniometer demonstrated restricted knee flexion and extension (Table 1). Patellofemoral and tibiofemoral joint mobility were markedly reduced (Table 2) [4]. Manual muscle testing revealed decreased strength in the affected lower limb musculature (Table 3) [5].

**Table 1:** Goniometric measurement of ROM (in degrees). ROM, range of motion

Joint	Movement	Preintervention	End feel	Postintervention	End feel
Knee (Left)	Flexion	0°-70°	Hard capsular	0°-105°	Hard capsular
	Extension	70°-0°	Hard	105°-0°	Hard

**Table 2:** Joint play gradings. 0, no movement; 1, considerable hypomobility; 2, slight hypomobility; 3, normal; 4, slight hypermobile; 5, considerable hypermobile; 6, complete instable

Joint	Direction	Preintervention	Postintervention
Knee (Left)	Anterior	1	2
	Posterior	1	2
	Lateral, medial	2	3
Patella (Left)	Inferior, superior, medial, and lateral	1	3

**Table 3:** Modified Medical Research Council manual muscle testing grading. 3-, gradual release from test position; 3+, holds test position against slight pressure; 4, holds test position against moderate pressure; 4+, holds test position against moderate to strong pressure; 5, holds test position against strong pressure

Joint	Movement	Preintervention	Postintervention
Knee (Left)	Flexors	3-	4+
	Extensors	3+	5
Hip (Left)	Flexors	3+	5
	Extensors	3	5
	Abductors	3-	4
	Adductors	3-	4
	Internal rotator	4	5
	External rotator	4	5

On examination, moderate tightness was noted in the left quadriceps and iliopsoas muscles. A 15-degree extensor lag and increased Q-angle were present. Knee proprioception was impaired (Table 4) [6].

**Table 4:** Extensor lag and knee proprioception.

On examination	Preintervention	Postintervention
Extensor lag (Left)	15°	0°
Knee proprioception (Left)	Affected by >5°	Normal

Investigation 

**Figure 1:**
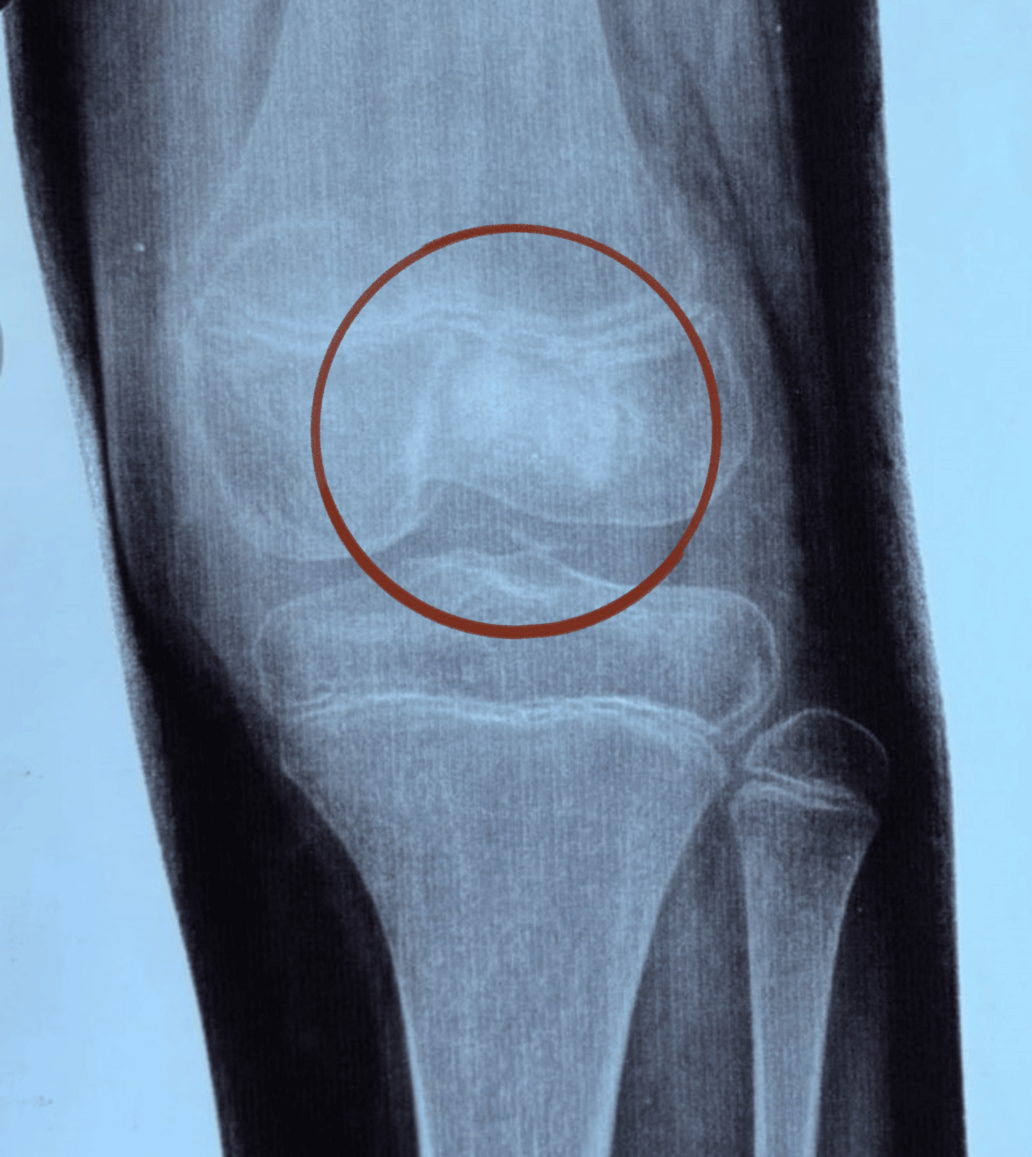
X-ray of the left knee. Anteroposterior (AP) view showing an irregular intramedullary mixed lytic-sclerotic lesion (red circle) in the distal femur.

**Figure 2:**
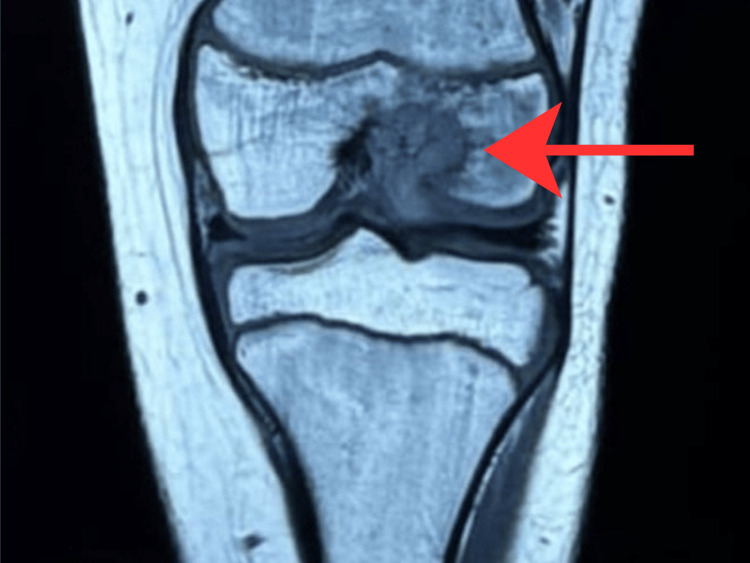
MRI of the left knee. An osseous neoplastic lesion (red arrow) located at the medial aspect of the lateral femoral condyle, extending to the adjoining intercondylar roof with intra-articular involvement, suggestive of osteoblastoma. Minimal interstitial edema was noted in the anterior cruciate ligament, with fibers remaining intact. MRI, magnetic resonance imaging

**Figure 3:**
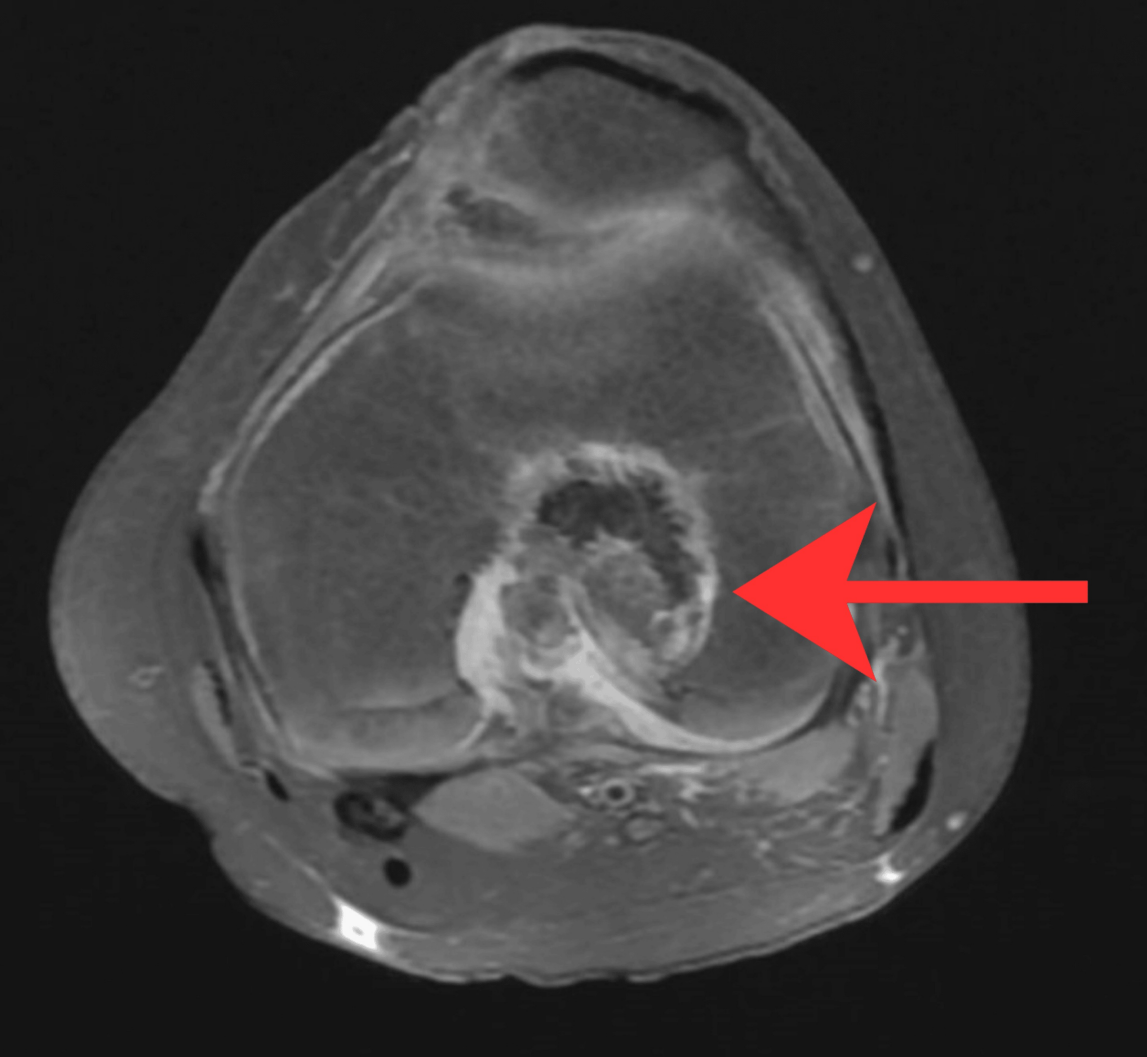
Immediate postoperative MRI of the left knee. Mucoid degeneration of the anterior cruciate ligament and significant dystrophic calcification within the posterior femoral intercondylar notch (red arrow). MRI, magnetic resonance imaging

Preoperative imaging included X-ray (Figure 1) and MRI (Figure 2). The patient subsequently underwent intra-articular curettage with bone grafting. Histopathological examination revealed a dense infiltrate of foamy histiocytes, lymphocytes, plasma cells, and polymorphonuclear leukocytes. The histiocytes showed characteristic emperipolesis. Immunohistochemistry was positive for S-100, OCT2, and cyclin D1, and negative for CD1a, Langerin, BRAF V600E, and ALK, consistent with intraosseous RDD. A postoperative MRI was performed (Figure 3).

Physiotherapy management

Due to the lack of specific literature on rehabilitation following neoplastic postoperative knee surgeries in the pediatric population, the physiotherapy protocol was self-developed by the authors with reference to available postoperative knee surgery rehabilitation protocols and was modified according to the patient’s functional status.

**Figure 4:**
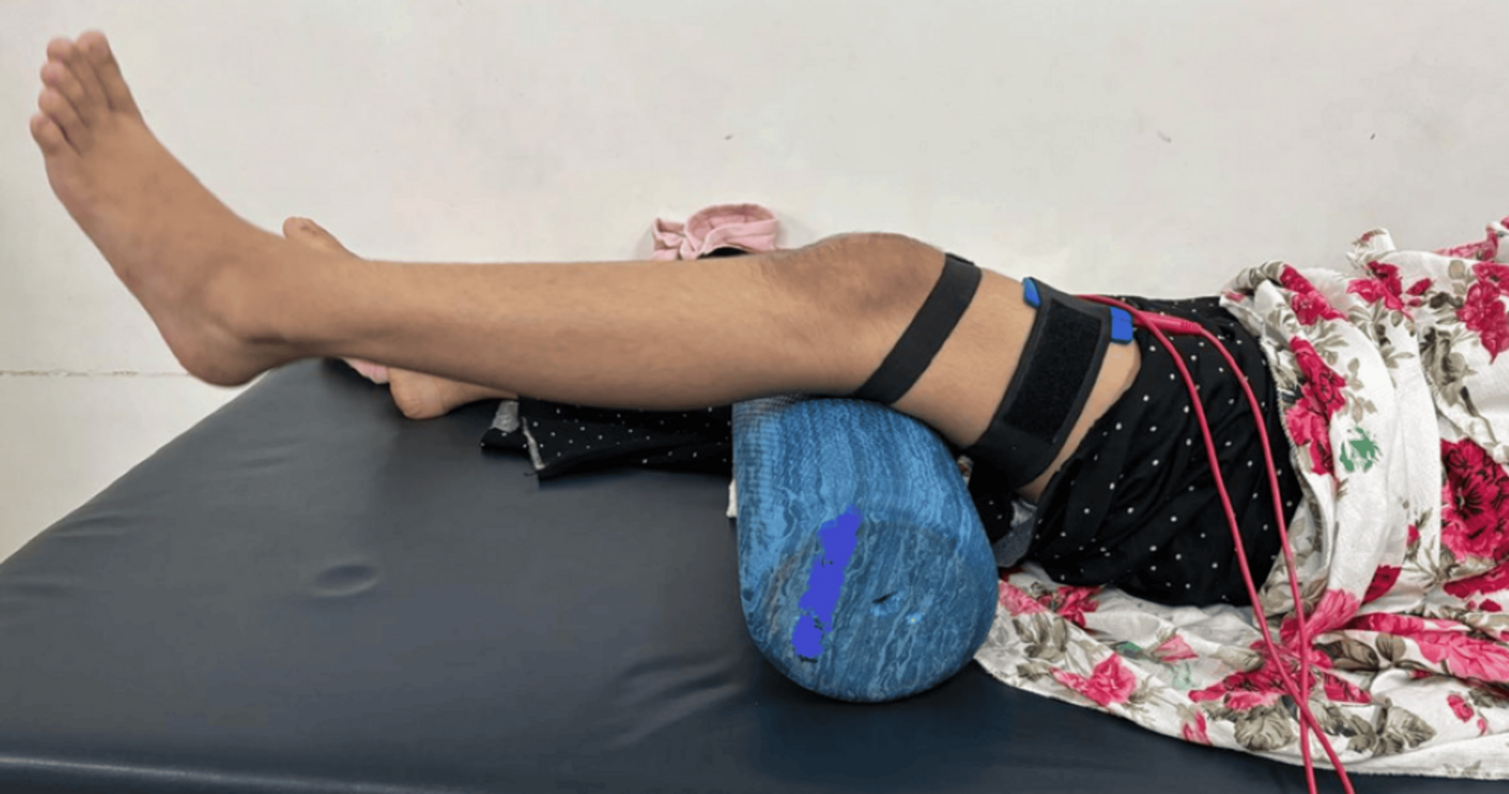
Surged faradic stimulation for VMO. VMO, vastus medialis oblique

**Figure 5:**
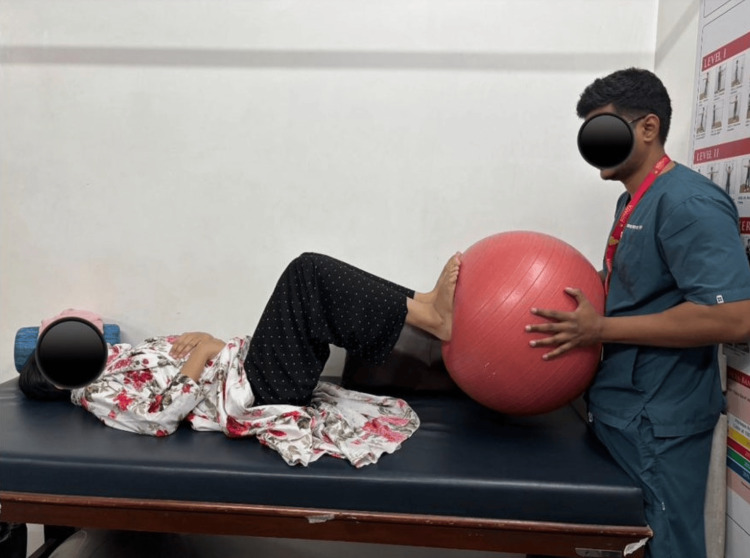
Patient performing ball press exercise.

The physiotherapy rehabilitation program was planned over a 12-week period, beginning on day 1 (the day of admission for physiotherapy), and was divided into three phases. Each phase was clearly outlined with specific goals and criteria for progression (Table 5). Figures 4-5 show the patient receiving physiotherapy treatment. 

**Table 5:** Phase-wise physiotherapy management. ROM, range of motion; MET, muscle energy technique; PIR, post-isometric relaxation; VMO, vastus medialis oblique; PWB, partial weight bearing; FWB, full weight bearing; EO, eyes open; EC, eyes closed; ADL, activities of daily living

Phase	Goals	Intervention	Dosage	Rationale
Phase 1 (day 1 to 4 weeks)	Patient education	Education regarding the condition and rehabilitation phases		
	To achieve 0°-90° knee flexion ROM	MET-PIR [7]	3 reps with 7 seconds hold × 5 sets	PIR helps to prevent reflex inhibition, facilitating safe improvement in joint mobility
		Heel slides with overpressure by the belt	10 reps	Encourages gradual knee flexion ROM
	To improve strength	Surge faradic for VMO	30 reps with 3 sets	Enhances neuromuscular recruitment of quadriceps, preventing extensor lag
		Straight leg raises, hip abduction, hip extension, clamshell	10 reps with 5 seconds hold	To increase and maintain the muscle strength of the hip, knee, and ankle
		Ankle musculature strengthening with a yellow Thera band	10 reps with 5 seconds hold	
	To improve the gait pattern	Progress from PWB to FWB		Gradual loading restores normal gait mechanics
		Lateral and forward weight shifts	10 reps × 1 sets	Promotes early weight acceptance
	To improve neuromuscular control and proprioception	Double-leg stance, balance eyes open, progress to eyes closed	30 seconds × 1 rep	Stimulates proprioceptive feedback and postural control
	To improve ADLs	Sit to stand, step up, step down	10 reps × 2 sets	Restores basic ADL function
Progression criteria for phase 2	Minimal pain, ROM 0°-90°, no extensor lag, good quadriceps activation, ambulation with FWB			
Phase 2 (4-8 weeks)	To improve strength	Multiple-angle isometrics for the quadriceps muscle	5 reps with 5 seconds hold	Improves quadriceps strength throughout different joint angles
		Single-leg bridging	10 reps × 1 set	Enhances hip and core stability, contributing to knee control
	To improve proprioception of the knee, hip, and ankle joints	Ball press	10 reps with 5 seconds hold	Closed-chain strengthening improves joint stability with reduced shear forces
		Wall squats at 90 degrees		
		Mini lunges		
		Cycling	5 minutes	
	To improve balance	Tandem standing EO, EC on blue stability trainer	3 reps with 30 seconds hold	Challenges to postural stability and sensory integration
		Single leg stance on a stable surface		
	To improve gait	Treadmill	10 minutes	Facilitates symmetrical gait pattern and endurance
Progression criteria for phase 3	Able to perform pain-free closed chain exercises, 10 controlled squats to 90°, symmetrical gait, reciprocal stair climbing			
Phase 3 (8-12 weeks)	Balance	BOSU ball balance, tandem walking	2 rounds	Challenge dynamic stability
	Agility	Fast walking → brisk walking → light jogging	10 minutes daily	Gradual reintroduction to higher functional demands
		*Z* walking	2 rounds	
		Cone drills		
		Agility ladder		
	Plyometric	Double-leg mini hops	10 reps × 2 sets	Improves speed, coordination, and neuromuscular responsiveness
		Step-off landings (height 10 cm)		
		Toe tap quick alternation		
		Wall-supported mini hops		

Discharge criteria were achieved when the patient demonstrated pain-free performance of all functional activities, with no swelling or tenderness following walking or strengthening exercises. Muscle strength of the affected limb improved to approximately 85%-90% compared with the contralateral limb. The patient exhibited a normal gait pattern without any limp and was able to walk continuously for 30-45 minutes without pain. Functionally, the patient was able to perform 20 step-ups and step-downs and climb stairs reciprocally without pain or difficulty, indicating adequate strength, endurance, and functional independence for safe discharge from physiotherapy.

Functional outcome measure

Clinical outcome measures were used to assess improvement pre- and post-rehabilitation, as shown in Table 6. The Revised Musculoskeletal Tumor Society Rating Scale [8], which assesses pain, function, emotional acceptance, supports, walking ability, and gait, was utilized for this purpose. The Pediatric Balance Scale (PBS) is a 14-item functional assessment evaluating static and dynamic balance in children through tasks, with higher scores indicating better balance and functional independence [9].

**Table 6:** Functional outcome measure.

Scale	Preintervention	Postintervention
Revised Musculoskeletal Tumor Society Rating Scale [8]	10/25	25/25
Pediatric Balance Scale [9]	35/56	56/56

The rehabilitation program was well tolerated, with no reports of excessive pain, fatigue, or discomfort during or after therapy sessions.

Following delayed initiation of physiotherapy, she progressed from walking with a limp and using assistive devices to walking independently without support. She achieved marked functional recovery, including improved knee flexion, pain-free movement, and the ability to sit cross-legged, squat, jump, and participate in sports. She has now returned to school and resumed all daily and recreational activities.

## Discussion

An 11-year-old girl experienced discomfort, decreased knee range of motion, muscle weakness, altered gait, impaired proprioception, and fear of falling following extended immobilization and intra-articular involvement after surgical curettage and bone grafting. In this case, a phased, systematic physiotherapy program was implemented to address these limitations and protect the healing graft.

Early controlled mobilization using active-assisted range of motion and muscle energy techniques facilitated the gradual restoration of knee mobility while protecting the healing graft. Muscle energy techniques are appropriate in the early postoperative phase due to their ability to improve circulation and reduce soft tissue stiffness with minimal mechanical stress [7]. Progressive strengthening of the quadriceps and proximal hip musculature was emphasized to counter postoperative muscle inhibition and improve knee stability and gait control, consistent with existing orthopedic rehabilitation literature [10,11]. Balance and proprioceptive training were incorporated to address neuromuscular deficits and fear of falling, leading to improved postural control and confidence during ambulation [12].

Only after achieving sufficient strength, balance, and advanced functional capacity are agility and plyometric exercises implemented. In line with rehabilitation guidelines suggested for lower limb orthopedic and oncologic conditions, gradual exposure to higher-level functional tasks enabled a safe return to everyday and community activities [13].

For pediatric patients to achieve optimal postoperative recovery, general suggestions and precautions must be followed in addition to a structured rehabilitation program. Protection of the healing tissues should be the primary focus throughout rehabilitation. This may be achieved through appropriate weight-bearing progression, regulated loading, use of assistive devices when necessary, and patient and caregiver education to ensure compliance. A low-load, gradual progression approach should be preferred over excessive loading and aggressive stretching to prevent joint stiffness, muscle weakness, and neuromuscular deficits. Early initiation of controlled rehabilitation is also beneficial in promoting circulation and facilitating healing. To guide progression appropriately, therapists must continuously monitor pain, effusion, range of motion, and functional response [14].

A key limitation of this case is the absence of disease-specific rehabilitation literature and the inability to restore knee flexion beyond the functional range. Nevertheless, meaningful improvements in pain, strength, mobility, balance, and function highlight the value of structured rehabilitation and underscore the need for further research in rare osseous conditions.

## Conclusions

Surgical management combined with early, structured physiotherapy formed an integral part of the postoperative care plan. A phase-wise rehabilitation approach was implemented to facilitate the progressive restoration of knee mobility, muscle strength, balance, and gait, with observable improvements noted in functional outcome measures over the rehabilitation period.

Despite substantial gains, complete knee flexion could not be achieved due to persistent mucoid degeneration of the anterior cruciate ligament. The rehabilitation program was designed to protect the healing bone graft while restoring mobility. This case highlights the importance of early, individualized physiotherapy in rare postoperative osseous conditions.
